# Genomic-Assisted Marker Development Suitable for *CsCvy-1* Selection in Cucumber Breeding

**DOI:** 10.3389/fpls.2021.691576

**Published:** 2021-08-18

**Authors:** Erdem Kahveci, Zübeyir Devran, Ercan Özkaynak, Yiguo Hong, David J. Studholme, Mahmut Tör

**Affiliations:** ^1^M.Y. Genetik Tarim Tek. Lab. Tic. Ltd. Sti., Antalya, Turkey; ^2^Department of Plant Protection, Faculty of Agriculture, University of Akdeniz, Antalya, Turkey; ^3^Yüksel Tohum Tarim San. ve Tic. A. S., Antalya, Turkey; ^4^Research Centre for Plant RNA Signaling, College of Life and Environmental Sciences, Hangzhou Normal University, Hangzhou, China; ^5^Department of Biology, School of Science and the Environment, University of Worcester, Worcester, United Kingdom; ^6^Biosciences, College of Life and Environmental Sciences, University of Exeter, Exeter, United Kingdom

**Keywords:** CVYV, cucumber, marker assisted selection, kompetitive allele-specific PCR genotyping, plant breeding

## Abstract

Cucumber is a widely grown vegetable crop plant and a host to many different plant pathogens. *Cucumber vein yellowing virus* (CVYV) causes economic losses on cucumber crops in Mediterranean countries and in some part of India such as West Bengal and in African countries such as Sudan. CVYV is an RNA potyvirus transmitted mechanically and by whitefly (*Bemisia tabaci*) in a semipersistent manner. Control of this virus is heavily dependent on the management of the insect vector and breeding virus-resistant lines. DNA markers have been used widely in conventional plant breeding programs *via* marker-assisted selection (MAS). However, very few resistance sources against CVYV in cucumber exist, and also the lack of tightly linked molecular markers to these sources restricts the rapid generation of resistant lines. In this work, we used genomics coupled with the bulked segregant analysis method and generated the MAS-friendly Kompetitive allele specific PCR (KASP) markers suitable for *CsCvy-1* selection in cucumber breeding using a segregating F_2_ mapping population and commercial plant lines. Variant analysis was performed to generate single-nucleotide polymorphism (SNP)-based markers for mapping the population and genotyping the commercial lines. We fine-mapped the region by generating new markers down to 101 kb with eight genes. We provided SNP data for this interval, which could be useful for breeding programs and cloning the candidate genes.

## Introduction

Cucumber plants, *Cucumis sativus*, have been cultivated as a vegetable crop across the globe for centuries (Tatlioglu, 1993). The fruit is consumed as fresh or industrialized product, and the major producing countries are China (7,033,8971 tons), Turkey (1,916,645 tons), Russia (1,626,360 tons), Ukraine (1,034,170 tons), and Iran [871,692 tons (FAO, 2019)]. As an important vegetable, cucumber is challenged by many different fungal, oomycete, bacterial, and viral pathogens (Kong et al., 2015; Słomnicka et al., 2018; Bandamaravuri et al., 2020).

One of the most devastating viral pathogens is *Cucumber vein yellowing virus* (CVYV), which belongs to the *Potyviridae* family (Lecoq et al., 2000), has an RNA genome (Janssen et al., 2005), is transmitted mechanically and by whitefly, *Bemisia tabaci*, in a semipersistent manner (Mansour and Al-Musa, 1993), and infects a number of cucurbit species (Gil-Salas et al., 2011). The occurrence and heavy crop losses due to CVYV infection in the open fields and under protected cucumber crops have been reported in the Mediterranean countries from Israel to Portugal (Cohen and Nitzany, 1960; Louro et al., 2004). The main symptoms of CVYV on the cucumber include vein clearing followed by vein yellowing on the youngest leaves (Cohen and Nitzany, 1960), the occasional occurrence of yellow/green mosaics on the fruit (Cuadrado et al., 2007), and eventual general necrosis of the entire infected plant (Cohen and Nitzany, 1960). Mechanical transmission of the virus allows the use of cucumber as a test and indicative plant for multiplication.

*Cucumber vein yellowing virus* has been classified as a quarantine viral pathogen in the EPPO A2 Action List (https://www.eppo.int/ACTIVITIES/plant_quarantine/A2_list). Control of this virus relies heavily on the application of integrated pest management (IPM) practices that incorporate the ecosystem-based strategies, including cultural practices, biological and chemical control of the vector, and the use of resistant varieties (Horowitz et al., 2011). Sanitation, use of certified virus-free seedlings, and eradicating diseased plants parts are common practices for controlling viral plant pathogens (Hilje et al., 2001; Nazarov et al., 2020). Although chemical pesticides have been used to control the whitefly insect vector, concerns to human health, occurrence of insecticide resistance, and damage to the environment led to a search for alternative measures (Sani et al., 2020). Use of microbial biological control agents (MBCA), such as entomopathogenic fungi (Faria and Wraight, 2001; Sani et al., 2020), use of barrier or trap crops (Zhang et al., 2020), and use of beneficial insects, such as predators or parasitoids (Moreno-Ripoll et al., 2014), have been considered.

The RNA-guided genome editing using clustered regularly interspaced short palindromic repeats (CRISPR)-Cas9 has been also used to generate virus-resistant crops (Liu and Fan, 2014). For example, Chandrasekaran et al. (2016) used Cas9/sgRNA constructs to target the recessive *eukaryotic translation initiation factor 4E* (*eIF4E*) gene in cucumber. They reported that the homozygous T3 lines showed immunity to CVYV (Chandrasekaran et al., 2016) indicating the possibility of alternative new methods for CVYV control. Planting cultivars resistant to the whitefly and/or to the virus is one of the most important control measures in the CVYV management. In a study to identify cucumber lines resistant to whitefly, Novaes et al. (2020) screened 60 genotypes and found that accessions IAC-1214, IAC-1201, Campeiro, Japonês, IAC-1311, Kyria, and IAC-1175 displayed some low levels of attractiveness to these insects and suggested they could be included in the breeding programs to develop whitefly-resistant cucumber lines.

Genetics of resistance to CVYV have been investigated by several groups (Picó et al., 2003). A Spanish landrace of short cucumber, C. sat-10, was found to be monogenic and displaying dominant resistance to CVYV (Picó et al., 2008). Similarly, a cucumber cultivar named Kyoto-3-feet originating from Japan has been reported to be resistant to CVYV (Martín-Hernández and Picó, 2021); however, detailed information on the nature of these resistance mechanisms is not available. Cucumber hybrid lines resistant to CVYV exist in the commercial market; however, currently all the work for selecting resistant lines relies on traditional pathotesting efforts. Recently, Pujol et al. (2019) described their elegant study on the resistant accession CE0749, a CVYV-resistant long Dutch-type cucumber. They used genomics and bulked segregant analysis (BSA) (Michelmore et al., 1991) and fine-mapped a locus containing the gene *CsCvy-1* locus in a 625 kb region with 24 candidate genes (Pujol et al., 2019).

Here, we described our investigations on the identification of DNA markers for fine mapping *CsCvy-1* using genomics and BSA. We used both segregating F_2_ populations and the available commercial F_1_ hybrids, mapped the locus down to 101 kb with eight genes, and provided single-nucleotide polymorphism (SNP) data for the interval, which could be useful for plant breeding programs.

## Materials and Methods

### Plant Lines and Mapping Populations

An F_2_ mapping population, generated from a cross between a susceptible (YT-189-1) and a resistant (YT-MLN-33) cucumber inbred lines (Yüksel Tohum A.S., Antalya, Turkey), was used in the phenotyping and genotyping experiments. F_3_ families were raised by selfing the selected lines and used to determine the genotype of the F_2_ lines.

### Virus Isolate and Pathology Methods

The CVYV isolate used in this study was obtained from DSMZ (Braunschweig, Germany) and propagated in susceptible cucumber plants (*Cucumis sativus*, line YT-189-1). Virus inoculum was prepared by homogenizing 1 g infected leaves in 4 ml 0.01 M phosphate buffer (pH 7.0) containing 0.2% sodium sulfate and 0.2% diethyldithiocarbamic acid (DIECA, Sigma-Aldrich, St. Louis, MO, United States). After adding 600-mesh carborundum and active carbon, cotyledons of cucumber plants (parental lines, F_1_, and F_3_ generations), which were at the cotyledon to one-true-leaf stages, were mechanically inoculated. A second inoculation was performed 3 days after the first one to eliminate escapees. The inoculated cucumber seedlings were then kept in a growth chamber with temperature control set at 30/25°C (day/night) with a 16 h light/8 h dark photoperiod for 3 weeks and observed every 2 days. First symptoms were observed 5–7 days postinoculation (dpi), but became obvious after 12–15 dpi. After 3 weeks, no further symptom developments were observed; thus, 15 dpi was selected to be the optimal time for symptom evaluation. Plants showing clear symptoms including mosaic and vein yellowing on leaves were rated as susceptible, whereas those with no symptoms or a very light vein discoloration on only the oldest ones were accepted as resistant. A minimum of 20 plants was used per treatment.

### DNA Extraction and Genome Sequencing

Young leaves were collected from parental and F_2_ lines. Plant genomic DNA was isolated using the Wizard Magnetic Kit (Promega, Madison, WI, United States) following the instructions of manufacturer. DNA was extracted from each individual plant lines, and a gel electrophoresis was performed to determine whether high molecular weight DNAs were isolated. The resistant and susceptible bulks were generated from 20 resistant and 20 susceptible F_2_ individuals, respectively, as described in earlier studies (Devran et al., 2015, 2018). Genomic DNA library and sequencing have been carried out by the University of Exeter Sequencing Service after quality check of DNAs, generating 2 ×150 bp paired-end read data for each parent line and bulked (resistant and susceptible) pools with Illumina HiSeq 2500 (Illumina, Inc. San Diego, CA, USA).

### Analysis of Genomic Sequences

As previously described (Devran et al., 2018), we took the NGS analysis approach where the raw reads were trimmed using BBDuk (filter = 27, trimk = 27; https://sourceforge.net/projects/bbmap/) to remove Illumina adapters and to quality trim both ends to Q12. Subsequently, trimmed sequences from parental lines and the bulks were mapped onto the available reference cucumber genome (V2 and V3) using BBMap (https://sourceforge.net/projects/bbmap/), and the alignment data were converted to the BAM format (Li et al., 2009). As the *CsCvy-1* locus was previously mapped onto chromosome 5 (Pujol et al., 2019), the data from the interval on chromosome 5: 7,000,000–7,850,000 were extracted using SAMtools (Li et al., 2009). The variant detection has been performed using BCFtools (Li et al., 2009) and a publicly available custom script (https://github.com/davidjstudholme/SNPsFromPileups) as previously described; (Yemataw et al., 2018). Integrative Genomics Viewer (IGV) was used to visualize the alignment results (Robinson et al., 2011).

### Converting Single Nucleotide Variants to PCR-Based Markers

Several of the SNPs within the interval were converted to Kompetitive Allele Specific PCR markers (KASP) by taking 100 bases either side of the SNP. KASP primers were developed using the LGC's primer picker software, Middlesex, United Kingdom. The PCRs were performed in a total volume of 15 μl that included DNA (10 ng; 5 μl), KASP Assay Mix (0.2 μl), KASP Master Mix (7.5 μl), and distilled water (2.3 μl). The KASP assay reactions were performed using the LightCycler® 480 II (Roche) using 61–55°C touchdown protocol (https://biosearch-cdn.azureedge.net/assetsv6/KASP-thermal-cycling-conditions-all-protocols.pdf). The fluorescence signal was measured for 2 min at 25°C using a FluOstar Omega Microplate Reader (BMG LABTECH, Ortenberg, Germany).

### Confirming Interval and Identifying Marker-Assisted Selection (MAS)-Friendly Markers

As the *CsCvy-1* locus had been previously mapped (Pujol et al., 2019), we used some of the published KASP markers including CVYV-184, CVYV-187, CVYV-188, CVYV-190, and CVYV-122 in this work. Published and newly generated KASP markers were first tested on parents to confirm the identified polymorphisms and then 120 segregating F_2_ lines. Marker genotyping data and the viral disease phenotyping data were used to confirm the *CsCvy-1* interval. As we developed new markers (Supplementary Table 1) to narrow the interval down, we also tested these markers with the commercial F_1_ hybrid lines, which were obtained from the relevant companies. As F_2_ lines are a segregating population, markers discovered using F_2_s may not be a reliable MAS-friendly marker. Therefore, we used F_1_ hybrid lines to narrow the interval further down and identify the MAS-friendly markers.

### Genomic Sequences and Accession Numbers

Cucumber reference genome sequences ChineseLong 9930 v2 are at http://cucurbitgenomics.org/organism/2 ChineseLong 9930 v3 at (https://ftp.ncbi.nlm.nih.gov/genomes/genbank/plant/Cucumis_sativus/latest_assembly_versions/GCA_000004075.3_Cucumber_9930_V3/). The raw sequence reads aligning to the interval have been deposited in the Sequence Read Archive (SRA) and are accessible *via* BioProject accession PRJNA713378.

## Results

### Resistance to CVYV Segregates as a Single Locus

**Figure 1:**
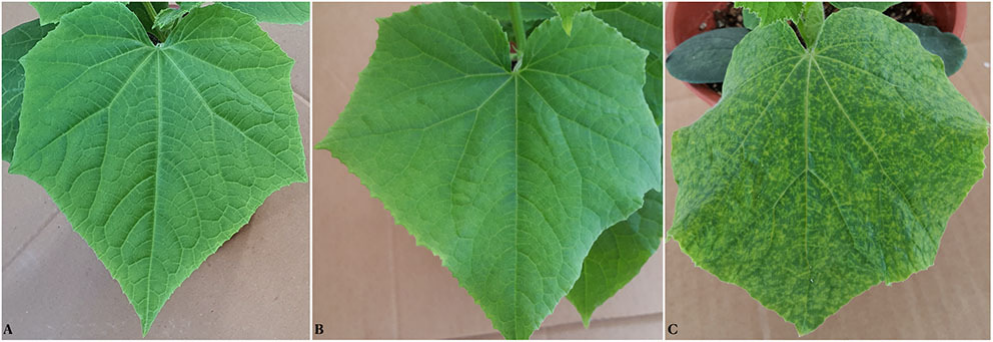
Healthy and *Cucumber vein yellowing virus* (CVYV)-infected cucumber leaves. Cucumber plants were mechanically inoculated at the cotyledon to one-true-leaf stages. A second inoculation was performed 3 days after the first one. The inoculated cucumber seedlings were then kept in a growth chamber with temperature control set at 30/25°C (day/night) with a 16 h light/8 h dark photoperiod for 3 weeks and observed every 2 days. The plants were evaluated for symptom development at 15 days after the first inoculation (dpi). Control plants were treated with buffer without virus in a similar manner. **(A)** Leaf of an uninoculated control plant, **(B)** leaf of a *Cucumber vein yellowing virus* (CVYV)-inoculated-resistant plant, and **(C)** leaf of a CVYV-inoculated-susceptible plant.

A cross was generated between the susceptible *C. sativus* inbred line YT-189-1 and the resistant inbred line, YT-MLN-33. The F_1_ hybrid showed resistance to CVYV, indicating that resistance was dominant. The F_1_ was selfed to generate segregating F_2_ populations. A total of 120 F_2_ lines were taken to F_3_ level, and 20 F_3_ lines descending from each F_2_s were inoculated with the virus to determine accurately the phenotype of the mapping population. Disease symptoms, including mosaics and vein yellowing, were obvious on the leaves of susceptible plants at 15 dpi (Figure 1). The segregation ratio observed in this bioassay was 92:28 (resistant:susceptible, 3:1; with Chi-square = 0.05 and *p* ≤ 0.05), suggesting that a single locus was providing resistance to CVYV in this cross and allowing the subsequent analysis.

### Linkage to *CsCvy-1* Locus

We used a next-generation sequencing (NGS)-based BSA approach whereby we generated bulks from DNA isolated from 20 resistant and susceptible F_2_ lines. We generated 150-bp paired-end Illumina HiSeq2500 sequencing data from the two parents and bulks (resistant and susceptible). A total of 390 million reads for each parent and 391 million reads for each bulk were generated. We then mapped these reads onto to the cucumber reference genome sequence (GenBank: GCA_000004075.3). However, during the course of our work, a locus designated *CsCvy-1* mapped on chromosome 5 was published using BSA approach (Pujol et al., 2019). This prompted us to check whether we were mapping the same region even though we were using different breeding lines. We used published CVYV-184, CVYV-187, CVYV-188, CVYV-190, and CVYV-122 KASP markers (Pujol et al., 2019) to determine whether the resistance locus in our parental line YT-MLN-33 is linked to *CsCvy-1*. Our mapping data showed a clear linkage (Table 1, Supplementary Figure 1), and therefore we concentrated on chromosome 5. As we had already performed an SNP analysis using the then-available version of the reference genome sequence (GCA_000004075.2), we developed several KASP markers and mapped the *CsCvy-1* locus in our segregating mapping population. To make our work comparable with the published data, we then mapped our clean NGS reads onto the updated reference genome sequence (GCA_000004075.3), concentrated on a region between SNP10218, identified in this work, and the published CVYV122 marker (Supplementary Table 2). Several of the published markers were not polymorphic for the parental lines we used, e.g., CVYV-173, CVYV-174, CVYV-175, and CVYV 176.

**Table 1:** Molecular markers used to define the interval for *CsCvy-*1 locus and the critical recombinant F_2_ lines.

F_**2**_ lines^*^	**10218**	**10317**	***CsCvy-1***	**10644**	**10950**	122^**^
58	Rr	Rr	Rr	**SS**	**SS**	Rr
59	RR	RR	RR	**Rr**	**Rr**	**Rr**
65	Rr	Rr	Rr	**RR**	**RR**	**Rr**
80	**Rr**	RR	RR	RR	RR	RR
103	**Rr**	**Rr**	RR	RR	RR	RR

* *F_2_ lines were generated from the cross between the resistant and the susceptible cultivars. **SS**, homozygous for susceptible parent allele; **RR**, homozygous for resistant parent allele; **RS**, heterozygous. Recombinants are shown in bold*.

** *This marker is from Pujol et al. (2019)*.

### Narrowing the Interval Using Nucleotide Variants

**Figure 2:**
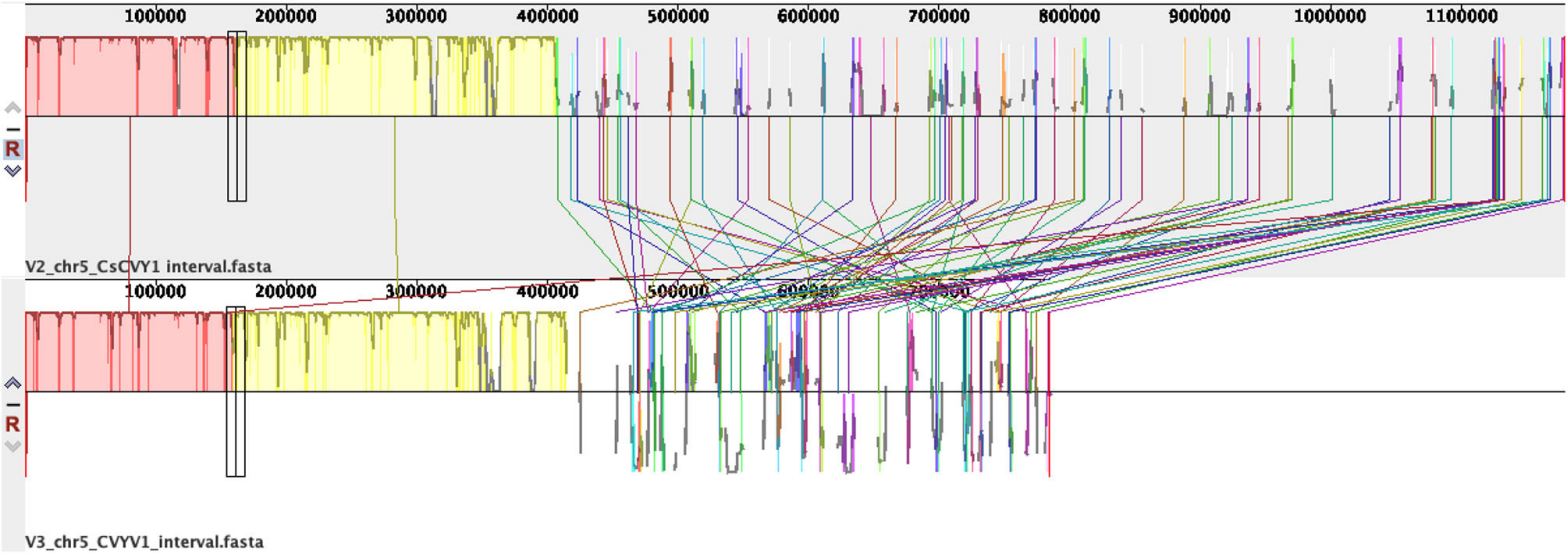
Pairwise sequence alignment of *CsCvy-1* interval in reference genomes version two and three (GCA_000004075.2 and GCA_000004075.3). Sequences were aligned using Progressive Mauve (Darling et al., 2004).

As our ultimate aim was to identify a marker that is tightly linked to *CsCvy-1*, we wanted to narrow the interval and generate further markers to identify an MAS-friendly marker. Using NGS data from parents and bulks, we mined the data on chromosome 5: 10,218,000–11,370,000 (ChineseLong 9930 ASM407v2, Supplementary Figure 1). KASP markers were then designed and used for mapping to narrow the interval. A total of 13 new KASP markers were generated, and the locus was mapped down to a 327-kb interval between the markers 10,317 and 10,644 K using the available F_2_ lines (Supplementary Table 2). As the version-three reference genome sequence became available, we also used this version and mined the data on chromosome 5: 7,000,000–7,850,000 (GCA_000004075.3, Supplementary Figure 2) for SNPs. A total of 436 SNPs have been detected (Supplementary Table 3). It should be noted here that although markers developed in this work using the GCA_000004075.V2 reference map to the region, several of the newly developed ones, especially toward the marker CVYV-122, were missing when the GCA_000004075.V3 reference was used. This may have been due to misassembly of the region as there was a 394 kb was missing in the GCA_000004075.3 genome (Figure 2).

### Commercial Varieties Help Narrowing the Interval

Although we had enough number of markers to map the locus further, the number of F_2_ lines to bring the interval down was not sufficient to identify further recombinants. We then obtained seeds of more than 20 commercial cucumber varieties with claimed CVYV phenotype and confirmed their phenotype by testing them with the CVYV isolate. Their DNAs were screened with our newly developed markers, and we narrowed down the locus to a 101-kb interval between the markers 10,317 and 10,418 K (Supplementary Table 4, Table 2). This finding suggests that the polymorphism identified in this work has been maintained across different varieties that have been used in the commercial breeding programs. In addition, the identified polymorphisms within the interval could be used in a breeding program by checking the existence of polymorphisms in the lines used.

**Table 2:** Molecular markers used to define the interval for *CsCvy-1* locus using F_1_ hybrids.

**F_**1**_**	**10270**	**10290**	**10317**	CsCvy-1^**^	***10418***	184^***^	**10644**	**10950**	122^***^
hybrids^*^								
Civan	Rr	Rr	Rr	R^****^	**SS**	**SS**	**SS**	**SS**	**SS**
Quinton	SS	SS	SS	SS	SS	SS	**RR**	SS	SS
Botanik	**Rr**	**Rr**	**Rr**	SS	SS	SS	**RR**	**Rr**	**Rr**
Kitir	SS	SS	SS	SS	SS	SS	**RR**	SS	SS
52-23	SS	SS	SS	SS	SS	SS	**RR**	SS	SS
Quarto	SS	SS	SS	SS	SS	SS	**RR**	SS	SS
Kybele	**Rr**	**Rr**	**Rr**	SS	SS	SS	**RR**	SS	SS

* *These were selected from more than 21 readily available varieties on the market*.

** *Phenotype information has been obtained from the web sites of companies, which sell these varieties to growers and confirmed by pathotesting*.

*** *These markers were from Pujol et al. (2019)*.

**** *The phenotype was not confirmed by using offspring to determine whether it is homozygous or heterozygous*.

*RR, Homozygous resistant; Rr, Heterozygous resistant; SS, Homozygous susceptible. Recombination is shown in bold*.

### The *CsCvy-1* Interval Contains Genes That May Play a Role in Defense

*CsCvy-1* locus mapped by Pujol et al. (2019) contained 24 genes. However, as we mapped the interval down to 101 kb, we used the annotations of the cucumber reference genome (GCA_000004075.3) to identify genes within the interval. The *CsCvy-1* locus in our mapping interval contains eight predicted genes (Table 3). Although *CsaV3_5G011160* encodes a cytochrome P450-like protein, *CsaV3_5G011170, CsaV3_5G011190, and CsaV3_5G011230* encode unknown proteins. However, *CsaV3_5G011180* encodes a serine/arginine repetitive matrix protein 2 isoform X2, *CsaV3_5G011220* encodes an endo-1,4-beta-xylanase, and two genes, *CsaV3_5G011200* and *CsaV3_5G011210*, both encode RNA-dependent RNA polymerase 1-like (RDR1-like) proteins. Interestingly, the deletion in the intragenic region of the RDR1 reported in this interval (Pujol et al., 2019) has been maintained in the resistant inbred line we used.

**Table 3:** Genes within the *CsCvy-1* interval in the Chinese Long cucumber genome.

**Gene ID**	**Putative function**
*CsaV3_5G011160*	Cytochrome P450-like protein
*CsaV3_5G011170*	Unknown protein
*CsaV3_5G011180*	Serine/arginine repetitive matrix protein 2 isoform X2
*CsaV3_5G011190*	Unknown protein
*CsaV3_5G011200*	RNA-dependent RNA polymerase 1-like
*CsaV3_5G011210*	RNA-dependent RNA polymerase 1-like
*CsaV3_5G011220*	Endo-1,4-beta-xylanase
*CsaV3_5G011230*	Unknown protein

## Discussion

Here, we present genetic evidence that a single dominant locus *CsCvy-1* confers resistance to CVYV infection in our inbred lines, consistent with a recent report (Pujol et al., 2019). Our genomic and molecular investigations using an F_2_ mapping population and also commercial resistant and susceptible varieties enabled us to map this locus down to 101-kb interval in which eight genes reside. Use of genomics allowed the identification of SNPs that could be used in breeding programs.

The plant immune system has the ability to recognize extracellular or intracellular molecules derived from plant pathogens and generate a defense response to restrict the pathogen growth or replication (Wang et al., 2008; Tör et al., 2009; Steinbrenner et al., 2015). Map-based studies usually involve the phenotyping and genotyping of a large number of individual plants in a segregating population. Using this approach, genes conferring resistance to fungal, oomycete, bacterial, and viral pathogens have been mapped, and many of them have been cloned (Tai et al., 1999; Borhan et al., 2008; Kim et al., 2017; Chen et al., 2021).

Linkage analysis plays a significant role in the cloning genes or generating markers tightly linked to the locus of interest using map-based approach. When we started this investigation, there was no published data on the chromosomal location of the gene conferring resistance to CVYV. During our SNP analysis, Pujol et al. (2019) published their work on the mapping of *CsCvy-1* in cucumber using genomic approach. We used relevant markers from this published work; however, several of them were not polymorphic in our parental lines, indicating the importance of generating SNP data from the lines used in generating mapping populations. After establishing the linkage between the resistance source in our material and the *CsCvy-1*, it was obvious that we were mapping the same locus, and thus we zoomed into the region.

We used genomics and BSA previously to clone genes (Woods-Tör et al., 2018) and to generate MAS-friendly molecular markers (Devran et al., 2018) that are tightly linked to the gene of interest. Our experience shows that although the use of reference genomes helps the identification of variants in the region of interest, different versions of reference genome assemblies produced different results in the SNP analysis. It was the case in this study where we initially used version 2 (GCA_000004075.2) as the reference and generated markers for our mapping work. Although all the markers generated from version 2 mapped the gene, several of them were missing when version 3 (GCA_000004075.3) were used, indicating the importance of mapping for confirmation and using more than one available reference genome.

High number of individual lines in a map-based study help identify the recombinant lines, which enables narrowing the interval. It can be easy to generate large number of F_2_ lines from plants, such as *Arabidopsis thaliana* (Tör et al., 2002). However, in plants such as cucumber, it may not be possible to achieve large number of F_2_s. In this work, we relied on 120 F_2_ lines to narrow the interval down to a 327 kb. Considering the breeding efforts where many characters are collected in a “pure” line, during which many crosses are carried out and many recombination events take place, for an MAS-friendly marker, the interval needs to be very small so that the likelihood of a recombination event between the marker and the gene of interest is almost zero. Bearing this in mind, we used the commercial cucumber F_1_ hybrids in our phenotyping and genotyping assays and reduced the interval down to 101 kb with eight genes.

Resistance to plant pathogens could be provided by membrane-bound proteins, such as receptor-like proteins (RLPs) (Wang et al., 2008) or receptor-like kinases (RLKs) (Roux et al., 2011; Zhang et al., 2013) or by the cytoplasmic nucleotide-binding, leucine-rich repeat (NLR) immune receptors (Adachi et al., 2019). There were no classic RLP, RLK, or NLR-type genes in the 101-kb *CsCvy-1* interval. Pujol et al. (2019) looked into the small variants and structural variation in the locus and argued that *CsaV3_5G011180* encoding for serine/arginine repetitive matrix protein (SARMP) could be a possible candidate. In addition, Pujol et al. (2019) postulated that *CsaV3_5G011200* and *CsaV3_5G011210* encoding RDRs 1a and 1b had the most appealing modifications in the locus and discussed the role of RDRs in RNA silencing pathways. Leibman et al. (2018) carried out detailed investigations into the *RDR1*-like genes in cucumber and reported the presence of four putative *RDR1*-family genes. They then investigated the expression of these *RDR1*-like genes and their role in defense against different viruses, including *Zucchini yellow mosaic virus* (ZYMV), CMV, and CVYV and showed that the level of *RDR1*-like gene expression varied according to the virus used (Leibman et al., 2018).

The NLR-type disease resistance genes in *Arabidopsis* have been reported to be clustered in the genome (Holub, 2007), and some of them function together and could be in head-to-head orientation, termed paired NLRs (Saucet et al., 2015). Further detailed studies indicated that one of them could function as a pathogen sensor, and the other member as signaling executor (Van de Weyer et al., 2019). Here, we have *RDR1a* and *RDR1b* in the interval right next to each other, functioning “like an *R*-gene” (Leibman et al., 2018), but it is not totally clear from expression studies whether they function together as some genetic investigations are needed. It is tempting to speculate that *RDR1a* and *RDR1b* are the most suitable candidate genes for the CVYV resistance.

Our strategy to use genomics and BSA to identify SNPs and generate molecular markers that could be employed in the selection of *CsCvy-1* enabled us to screen several markers and narrowed the interval down. These SNPS and markers could be used to identify polymorphism in different backgrounds in any breeding program to select *CsCvy-1*. Subsequent experiments could be designed to silence both *RDR1 and RDR2* genes individually and together in the same background to reveal their dependence onto each other and their contribution to CVYV defense.

## Data Availability Statement

The datasets presented in this study can be found in online repositories. The names of the repository/repositories and accession number(s) can be found at: https://www.ncbi.nlm.nih.gov/genbank/, PRJNA713378.

## Author Contributions

EK, ZD, and MT planned and designed the research. EK carried out pathology tests. ZD performed genotyping using markers. EÖ made crossings between plant lines and produced offspring. YH, MT, and DS analyzed and interpreted the data. EK, ZD, YH, DS, and MT wrote the manuscript. All authors contributed to the article and approved the submitted version.

## Conflict of Interest

EK was employed by company M.Y. Genetik Tarim Tek. Lab. Tic. Ltd. Sti. EÖ as employed by company Yüksel Tohum Tarim San. ve Tic. A. S. The remaining authors declare that the research was conducted in the absence of any commercial or financial relationships that could be construed as a potential conflict of interest.

## Publisher's Note

All claims expressed in this article are solely those of the authors and do not necessarily represent those of their affiliated organizations, or those of the publisher, the editors and the reviewers. Any product that may be evaluated in this article, or claim that may be made by its manufacturer, is not guaranteed or endorsed by the publisher.

## Supplementary Material

Supplemental Figure 1 Position of SNP-based markers on chromosome 5 in ChineseLong 9930 ASM407v2.

Supplemental Figure 2 Position of SNP-based markers on chromosome 5 in ChineseLong 9930 GCA_000004075.3.

Supplemental Table 1 KASP marker sequences developed in this study and previously published work.

Supplemental Table 2 Mapping *CsCvy-1* with existing and newly generated KASP markers using F_2_ population.

Supplemental Table 3 SNPs and their positions in *CsCvy-1* locus on chromosome 5 using GCA_000004075.3. Proportions of aligned reads supporting the variant base are given.

Supplemental Table 4 Mapping *CsCvy-1* with existing and newly generated KASP markers using F_1_ hybrids.
