# Supplementary figures and images for: Genomic-Assisted Marker Development Suitable for CsCvy-1 Selection in Cucumber Breeding
Source: Front Plant Sci. 2021 Aug 18;12:691576. doi: 10.3389/fpls.2021.691576 (PMC8416629; doi:10.3389/fpls.2021.691576)

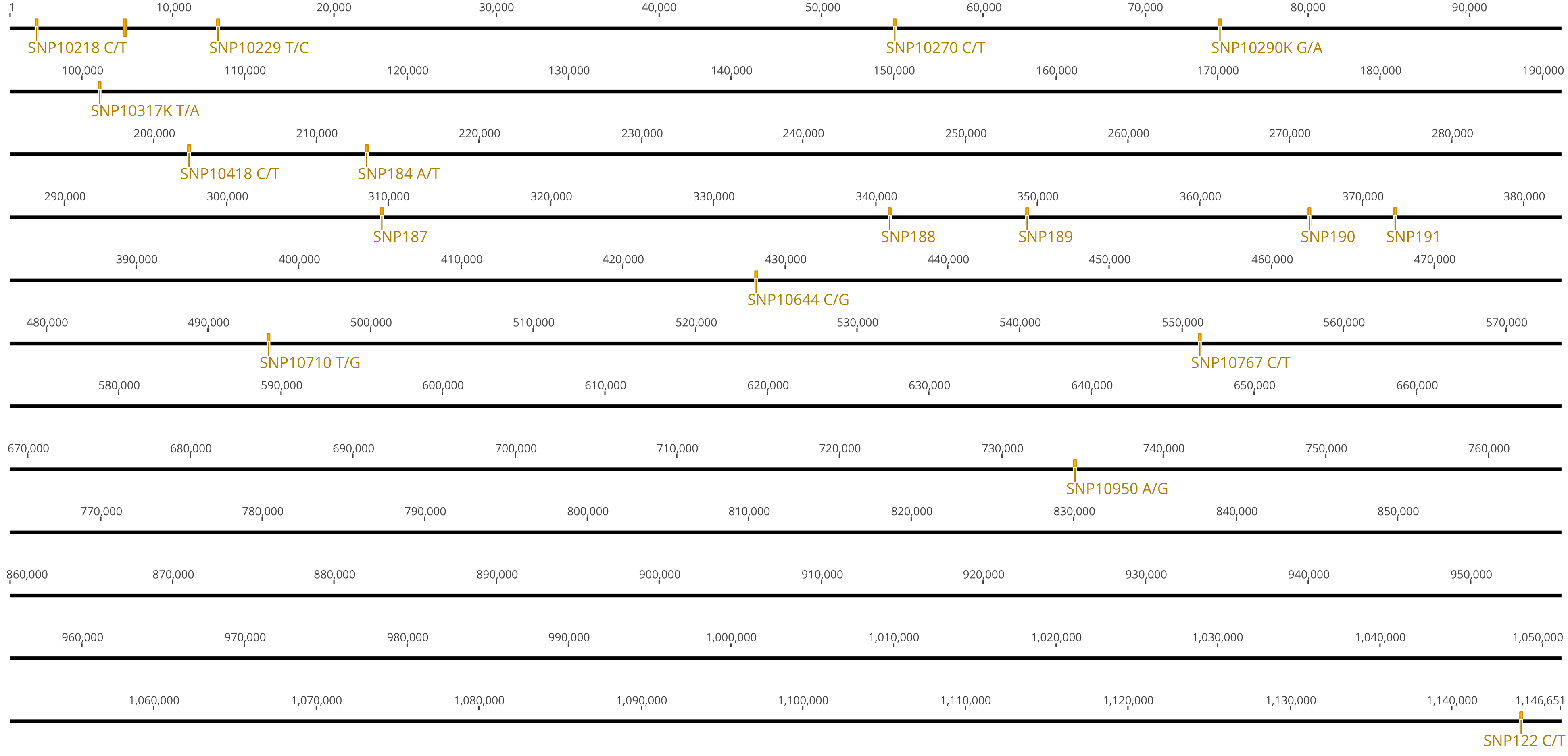

Supplement: Supplemental Figure 1 — Position of SNP-based markers on chromosome 5 in ChineseLong 9930 ASM407v2. [file Image_1.TIFF]

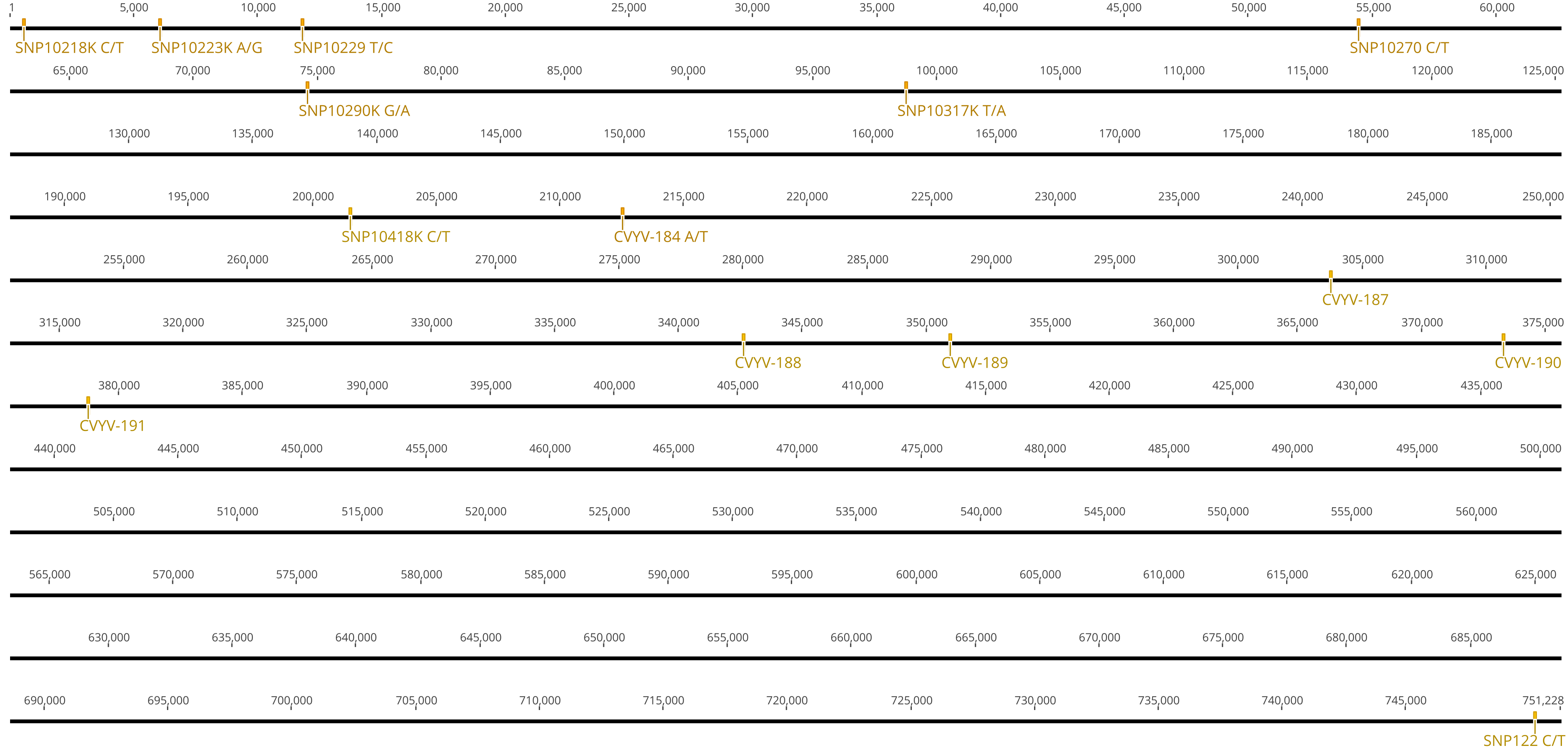

Supplement: Supplemental Figure 2 — Position of SNP-based markers on chromosome 5 in ChineseLong 9930 GCA_000004075.3. [file Image_2.TIFF]
